# Decreased Levels of Circulating IL17-Producing CD161^+^CCR6^+^ T Cells Are Associated with Graft-versus-Host Disease after Allogeneic Stem Cell Transplantation

**DOI:** 10.1371/journal.pone.0050896

**Published:** 2012-12-04

**Authors:** Anniek B. van der Waart, Walter J. F. M. van der Velden, Astrid G. S. van Halteren, Marij J. L. G. Leenders, Ton Feuth, Nicole M. A. Blijlevens, Robbert van der Voort, Harry Dolstra

**Affiliations:** 1 Department of Laboratory Medicine - Laboratory of Hematology, Radboud University Nijmegen Medical Centre, Nijmegen, The Netherlands; 2 Department of Hematology, Radboud University Nijmegen Medical Centre, Nijmegen, The Netherlands; 3 Immunology Laboratory, Department of Pediatrics, Leiden University Medical Center, Leiden, The Netherlands; 4 Department of Epidemiology, Biostatistics and Health Technology Assessment, Radboud University Nijmegen Medical Centre, Nijmegen, The Netherlands; University of Southern California, United States of America

## Abstract

The C-type lectin-like receptor CD161 is a well-established marker for human IL17-producing T cells, which have been implicated to contribute to the development of graft-versus-host disease (GVHD) after allogeneic stem cell transplantation (allo-SCT). In this study, we analyzed CD161^+^ T cell recovery, their functional properties and association with GVHD occurrence in allo-SCT recipients. While CD161^+^CD4^+^ T cells steadily recovered, CD161^hi^CD8^+^ T cell numbers declined during tapering of Cyclosporine A (CsA), which can be explained by their initial growth advantage over CD161^neg/low^CD8^+^ T cells due to ABCB1-mediated CsA efflux. Interestingly, occurrence of acute and chronic GVHD was significantly correlated with decreased levels of circulating CD161^+^CD4^+^ as well as CD161^hi^CD8^+^ T cells. In addition, these subsets from transplanted patients secreted high levels of IFNγ and IL17. Moreover, we found that CCR6 co-expression by CD161^+^ T cells mediated specific migration towards CCL20, which was expressed in GVHD biopsies. Finally, we demonstrated that CCR6^+^ T cells indeed were present in these CCL20^+^ GVHD-affected tissues. In conclusion, we showed that functional CD161^+^CCR6^+^ co-expressing T cells disappear from the circulation and home to GVHD-affected tissue sites. These findings support the hypothesis that CCR6^+^CD161-expressing T cells may be involved in the immune pathology of GVHD following their CCL20-dependent recruitment into affected tissues.

## Introduction

Graft-versus-host disease (GVHD) is still one of the major causes of morbidity and mortality after allogeneic stem cell transplantation (allo-SCT) [1]. The pathophysiology of GVHD is a multistep process involving tissue damage and pro-inflammatory cytokine cascades induced by the pre-transplant conditioning regimen [1], [2]. This results in an excessive inflammatory environment in which donor-derived CD4^+^ and CD8^+^ T cells become potently activated. In addition, T cell trafficking towards inflamed GVHD-prone organs including skin, lung, gastrointestinal tract, and liver is increased. Further tissue destruction in these organs occurs in the case of presentation of ubiquitous or epithelial expressed allo-antigens to infiltrating allo-reactive cytotoxic T cells. Moreover, macrophages, and other effector T cells subsets are recruited, resulting in further enhancement of GVHD [1], [3]–[5]. In particular, Th1-type CD4^+^ T cells and Tc1-type CD8^+^ T cells play an important role in GVHD pathophysiology [6], [7], but other T cell subsets with specific phenotype and functional characteristics might play a pivotal role as well.

The contribution of pro-inflammatory Th17 cells in various autoimmune disorders has raised the question of the role of IL17-producing T cells in GVHD. Although some mouse studies showed that these T cells are involved in the onset and persistence of GVHD [8]–[11], others claim a protective role of Th17 cells [12]. Furthermore, human studies performed on Th17 cells also show conflicting results [13]–[16]. An increase in circulating Th17 cells, as well as an imbalance between Th17 and regulatory T cells, has been correlated with occurrence of GVHD [13]–[15]. However, Broady *et al.* found an expansion of Th1 rather than Th17 cells in GVHD-affected skin [13]. Though, Bossard *et al.* recently showed that the absolute number of Th17 cells using the markers CD161, RORγt, and CC chemokine receptor (CCR)6 were significantly higher in intestinal mucosa of patient with acute GVHD [16]. Regarding the proposed plasticity of Th17 responses [17], CD161 and CCR6 may be more reliable surface markers to study the involvement of Th17 and Tc17 cells in GVHD.

Interestingly, it has been shown that CD161 is a distinguishing surface marker for both CD4^+^ and CD8^+^ T cell subsets producing IL17 and/or IFNγ [18], [19]. CD161, also known as killer cell lectin-like receptor superfamily B member 1 (KLRB1) or natural killer receptor protein 1a (NKRP1a), is a type II membrane glycoprotein with characteristics of the C-type lectin superfamily [20]. The function of CD161 on T cells has not been clearly defined yet, but a role in T cell co-stimulation has been indicated [21], [22]. Furthermore, CD161 has been implicated to play a role in trans-endothelial migration [23], [24]. CD161 is moderately expressed on CD4^+^ T cells, but within the CD8^+^ T cells population a distinct subset clearly expresses high levels of CD161 [25]–[32]. The comparable phenotype between Th17 cells and CD161^hi^CD8^+^ T cells, including explicit IL17-production and their expression of RORγt [25], [27], [30], suggests that these cells are the equivalent of Th17 cells within the CD8^+^ T cell population, namely Tc17 cells. Distinctively, CD161-expressing T cells display high levels of the chemokine receptor CCR6, for which we recently showed that a single nucleotide polymorphism in this gene correlates with occurrence of chronic GVHD [33]. CCR6 has only one ligand, CCL20, which is constitutively expressed in organs such as the liver, colon, small intestine, lung, and skin [34]. Furthermore, damage of the epidermal permeability barrier, as well as stimulation with IL1ß, results in up-regulation of CCL20 expression [35], [36]. This suggests that CD161-expressing T cells have the capability to migrate to the target organs involved in GVHD.

In this study, we investigated the repopulation kinetics, functional properties, and associations with GVHD of CD161-expressing CD4^+^ and CD8^+^ T cells in patients who received allo-SCT for a haematological malignancy. We observed that decreased levels of circulating CD161-expressing T cells, with the specific migratory capacity towards CCL20 expressed in GVHD tissues, and the potential to secrete IL17 and IFNγ, correlate with the occurrence of GVHD.

## Materials and Methods

### Patients and Healthy Controls

Patients treated between 1993 and 2008 with allo-SCT for a haematological malignancy in the Radboud University Nijmegen Medical Centre were included in this study (Table 1). All patients received a HLA-matched sibling allo-SCT which, irrespective of donor source or conditioning regimen, were partially depleted of T cells. Until November 2001 counterflow centrifugation elutriation was used [37], and from that moment onwards T cell depletion was performed with immunomagnetic CD34-enrichment or CD3/CD19 depletion (Miltenyi Biotec) [38]. CD3^+^ T cells were added back in the graft with a median ± SD of 0.43±0.51×10^6^ cells/kg bodyweight. GVHD prophylaxis consisted of Cyclosporine A (CsA) in almost all patients, and was dosed 1.5 mg/kg bidaily intravenously for the first two weeks, and thereafter 1 mg/kg bidaily intravenously or 2.5–3 mg/kg bidaily orally. Tapering of CsA was started in the absence of GVHD after two months and stopped at three months. The occurrence of acute GVHD was graded according to the criteria by Przepiorka *et al.* [39]. Chronic GVHD was classified according to the revised Seattle criteria of Lee *et al.* [40]. PBMCs were isolated using Ficoll-Hypaque gradient from PB samples that were collected at several time points after allo-SCT. Absolute cell levels were calculated using the lymphocyte count from the PB sample and the percentages of the different T cells populations measured in the PBMCs. PBMCs of healthy controls were isolated from buffy coats supplied by the Sanquin Blood Supply Foundation. Patients and healthy donors had given their informed consent which was approved by the RUNMC Institutional Review Board.

**Table 1:** Patient characteristics.

Characteristic	*n = 76*
*Age at transplantation, years (median, range)*	47 (19–63)
*Sex, no. males (%)*	42 (55%)
*Underlying disease, no. (%)*	
- ALL/AML/MDS	53 (70%)
- CML	11 (14%)
- NHL/CLL	12 (16%)
*Conditioning regimen, no. (%)*	
- Cy-Bus	4 (5,5%)
- Cy-TBI	10 (13%)
- Ida-Cy-Bus	7 (9%)
- Ida-Cy-TBI	55 (72,5%)
*Graft source*	
- Bone marrow	32 (42%)
- Peripheral Blood	44 (58%)
*Acute GVHD, no. (%)*	
- Grade 1–4	40 (53%)
- Grade 3–4	9 (12%)
*Chronic GVHD, no. (%)*	
- Limited	17 (22%)
- Extensive	14 (18%)

Abbreviations: ALL, acute lymphatic leukemia; AML, acute myeloid leukemia; MDS, myelodysplastic syndrome; CML, chronic myeloid leukemia; NHL, non-Hodgkin lymphoma; CLL, chronic lymphatic leukemia; Cy, ***cyclophosphamide; Bus, busulphan; TBI, total body irradiation***; ***Ida, idarubicin;*** GVHD, graft-versus-host disease.

### Flow Cytometry

Flow cytometry was performed using the following directly conjugated antibodies: CD3 (UCHT1, Beckman Coulter, Biolegend), CD4 (13B8.2, Beckman Coulter; OKT4, Biolegend), CD8 (3B5, Invitrogen), CD45RA (H100, Biolegend), CD161 (191B8, Miltenyi Biotech), CCR6 (53103, R&D Systems), CCR7 (150503, R&D Systems; G043H7, Biolegend). Expression of CD161 and additional markers was determined by staining for 30 minutes at 4°C. Cells were washed with phosphate-buffered saline (PBS)/0.5% BSA (Sigma). To determine the Rhodamine 123 (Rh123, Sigma) efflux, cells were labelled with 10 µg/ml Rh123 in IMDM containing 1% BSA for 30 minutes at 4°C. Then, cells were incubated for 30 minutes in the cell incubator at 37°C, followed by a standard staining procedure. Cells were measured on the Cyan-ADP 9 color analyzer (Beckman Coulter). Analysis was performed using Summit software.

### Quantative RT-PCR

Quantitative RT-PCR for expression of the ABCB1 transporter was performed on T cell subsets. From both CD4^+^ and CD8^+^ T cells, CD161-subsets were sorted on a FACS ALTRA (Beckman Coulter). Total RNA was extracted using the Quick-RNA miniPrep isolation kit (Zymo Research) in accordance with the manufacturer’s instructions. Subsequently, cDNA was generated using standard methods [41]. ABCB1 expression was determined using the following primers and probe: ABCB1-forward, 5′- ACTGAGCCTGGAGGTGAAGAAG-3′; ABCB1-reverse, 5′-TTTGCCATCAAGCAGCACTT-3′; ABCB1-probe, 5′-FAM-TCCTGGAGCGGTTCTACGACCCCTT-3′. Reactions were run on an ABI 7900-HT Real-time PCR System and analyzed with Sequence Detection Systems 2.3 software (Applied Biosystems). Expression is shown in ΔΔCt values and was quantified relative to CD161^−^CD4^+^ T cells, which was set at 1 ΔΔCt value. ΔΔCt was calculated as follows: 2^∧^(-ΔCt_sample_ − ΔCt_CD161_−_CD4+ T cells_), in which ΔCt was normalized for the porphobilinogen deaminase (PBGD) housekeeping gene by calculating ΔCt = Ct_ABCB1_ − Ct_PBGD_ per sample.

### T Cell Proliferation Assay

To compare the effect of immunosuppressive drugs on proliferation of CD161^hi^ versus CD161^neg/low^CD8^+^ T cells, PBMCs from healthy controls were labelled with 5 µM carboxyfluorescein diacetate succinimidyl (CFSE, Molecular Probes Europe) for 15 minutes at 37°C in IMDM. Cells were stimulated in a mixed lymphocyte reaction (MLR) using irradiated allo-PBMCs in IMDM (Invitrogen)/10% human serum (HS, PAA Laboratories), 200 U/ml IL2 (Chiron), 5 ng/ml IL7 (Immunotools), and 5 ng/ml IL15 (Immunotools). CsA (BioVision) was added at the start of the MLR culture. CFSE levels of CD161^hi^ and CD161^neg/low^CD8^+^ T cells were measured by flow cytometry after seven days.

### Intracellular Cytokine Staining

To determine the cytokine production by the different T cell subsets, intracellular cytokine staining was performed. PBMCs were stimulated with PMA and ionomycin for 4 hours with Brefeldin A (all BD) present for the last 3 hours. Cells were stained for CD3, CD4, CD8, and CD161 as described. Then cells were fixated, permeabilized (eBioscience), and stained for IL17 (eBioscience) and IFNγ (BD). Non-stimulated cells and IgG1 (Dako) stainings were used as controls.

### Immunohistochemistry

Enzymatic immunohistochemical staining for CCL20 was performed as described previously [42] on 4 µM formalin fixed paraffin embedded tissue sections prepared from skin and gut biopsies obtained from patients diagnosed with either acute or chronic GVHD. Briefly, after baking for 1 hour at 66°C, slides were subsequently deparaffinised in xylol and rehydrated in a series of ethanol. Endogenous peroxidase was blocked using methanol/0.3% H_2_0_2_ for 20 minutes. The sections were then subjected to heat mediated antigen retrieval in a microwave using 10 mM citrate buffer (pH 6.0). Anti-CCL20 (R&D Systems) was diluted in PBS/1% BSA and the sections were incubated overnight at RT in a humidity chamber. Anti-CCL20 was detected enzymatically using Powervision (Dako) followed by colour development with 3.3-diaminobenzidine. After counterstaining with haematoxylin, the sections were mounted in Pertex.

Triple immunofluoresent staining was performed in parallel on tissue sections that were subjected to deparaffinization and antigen retrieval as described above. Note that endogenous peroxidise blocking was not performed. Primary antibodies specific for CD3 (DAKO), CD4 (R&D Systems), and CCR6 (R&D Systems), were diluted in PBS/1% BSA and the sections were incubated overnight at RT in a humidity chamber. Bound antibodies were detected with appropriated diluted secondary antibodies (donkey anti-rabbit IgG Alexa-546, donkey anti-mouse IgG Alexa-488 and donkey anti-goat Alexa-647, all from Invitrogen). After washing in PBS, the sections were mounted in moviol.

### Migration Assay

Migration towards CCL20 was measured in 96 or 24 well plates containing transwell inserts with 5 µm pores (Costar). IMDM/10% HS with CCL20 (Immunotools) was added to the lower compartment. CD3^+^, CD4^+^ or CD8^+^ T cells were selected using the standard MACS procedure (Miltenyi Biotech) and 0.75 or 2.5×10^6^ cells were loaded into the inserts. After 2 hours in a 37°C incubator, cells in the lower compartment were harvested and labelled for CD3, CD4 or CD8, and CD161. Migration was determined by flow cytometry by acquiring events for a fixed time period and presented as the relative migration compared to input.

### Statistical Analysis

Statistical analysis for experimental data was performed using GraphPad Prism 4. Statistical significance of difference was analyzed using an one-tailed student *t-*test, parametric, non-parametric, one-way or two-way analysis of variance (ANOVA), followed by the appropriate post-hoc test, all as indicated in the figure legends. Correlation analysis was performed by calculating the Spearman correlation coefficient.

Associations of CD161^+^CD4^+^ and CD161^hi^CD8^+^ T cells with the occurrence of acute or chronic GVHD after allo-SCT were tested using univariate logistic regression analysis. To this end the percentages and absolute cell counts of CD161^hi^CD8^+^ levels, as well as the absolute levels of CD161^+^CD4^+^ T cells were logarithmically transformed because of their positive skewing. To correct for possible confounders, we used multivariate logistic regression analyses. Regression analyses were performed using SAS 8.2 software. For all analyses *P*<0.05 were considered to be significant.

## Results

### Determining the Normal Distribution of CD161-expressing T Cells in Healthy Controls

In order to evaluate the repopulation kinetics of CD161^+^CD4^+^ and CD161^hi^CD8^+^ T cells in allo-SCT recipients, we first determined their frequency and absolute numbers in peripheral blood (PB) of healthy controls. Within the CD4^+^ T cell population, a clear CD161^+^ subset could be identified, with an average ± SD of 17.7±5.8% and 174.1±47.8×10^6^ CD161^+^CD4^+^ T cells/L (Figure S1A–B, *n* = 16). As expression levels of CD161 were higher on CD8^+^ T cells, CD8^+^ T cells were divided in CD161^neg/low^ and CD161^hi^ cells (Figure S1C). An average of 11.6±7.2% (*n* = 42) CD161^hi^ cells within the CD8^+^ T cell population and 48.3±45.9×10^6^ (*n* = 22) CD161^hi^CD8^+^ T cells/L was observed (Figure S1D). Reference ranges were set as the mean ± SD to compare cell numbers found in allo-SCT recipients with those of healthy controls.

### Reconstitution of CD161-expressing T Cells after HLA-matched Allo-SCT

**Figure 1:**
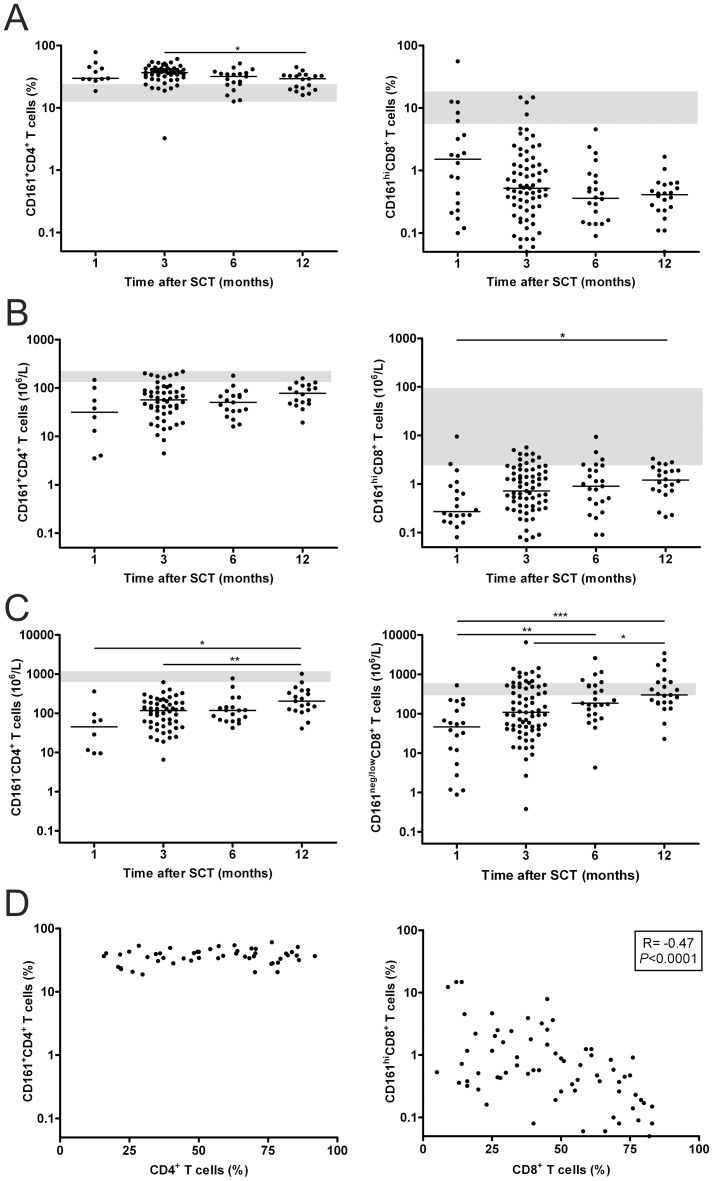
Reconstitution of CD161-expressing T cells in patients after allo-SCT. (A) Percentage of circulating CD161^+^ within CD4^+^ and CD161^hi^ within CD8^+^ T cells in patients at 1 (*n* = 11, 20), 3 (*n* = 50, 71), 6 (*n* = 19, 24), and 12 (*n* = 19, 22) months after allo-SCT. (B) Absolute levels of circulating CD161^+^CD4^+^ and CD161^hi^CD8^+^ T cells in patients at 1 (*n* = 8, 20), 3 (*n* = 50, 71), 6 (*n* = 19, 24), and 12 (*n* = 19, 22) months after allo-SCT. (C) Absolute number of circulating CD161^−^CD4^+^ and CD161^neg/low^CD8^+^ T cells in patients at 1 (*n* = 8, 20), 3 (*n* = 54, 69), 6 (*n* = 19, 24), and 12 (*n* = 19, 22) months after allo-SCT. (D) Correlation between the percentage of circulating CD161^+^CD4^+^ and CD4^+^ T cells (*n* = 58), and CD161^hi^CD8^+^ T cells and CD8^+^ T cells (*n* = 70) at 3 months after allo-SCT. Lines represent median value, grey areas represent the reference range of healthy controls (mean ± SD). Statistical analysis was performed using a One-way ANOVA followed by a Bonferroni post-hoc test (CD4) or non-parametric One-way ANOVA followed by a Dunns post-hoc test (CD8). Correlations were determined by calculating the Spearman correlation coefficient (R). *P<0.05, **P<0.01, ***P<0.001.

To investigate the repopulation kinetics of CD161-expressing T cells in patients after HLA-matched allo-SCT, PBMCs isolated from peripheral blood of 76 patients at 1, 3, 6, and 12 months post-transplant were analyzed by flow cytometric analysis. While the percentage of CD161^+^CD4^+^ T cells were found to be higher compared to healthy controls, the CD161^hi^CD8^+^ T cell population was lower (Figure 1A). Although in most patients CD161^hi^CD8^+^ T cells were present at low frequency, in some patients a relative high frequency of CD161^hi^CD8^+^ T cells could be detected at one month after allo-SCT. However, the frequency of both CD161^+^CD4^+^ as well as CD161^hi^CD8^+^ T cells diminished in time. However, CD161^+^CD4^+^ T cells mainly decreased from 3 months onwards, while the frequency of CD161^hi^CD8^+^ T cells already declined after one month where after it remained relatively stable (Figure 1A). On the other hand, the absolute number of CD161^+^CD4^+^ and CD161^hi^CD8^+^ T cells increased in the first months after allo-SCT, where after they stabilized but remained below the reference ranges (Figure 1B). Meanwhile, the total CD4^+^ and CD8^+^ T cell number expanded. From 3 months onwards, a relative overshoot of the percentage (data not shown) and absolute number of CD161^neg/low^CD8^+^ T cells was observed in a substantial proportion of the patients (Figure 1C).

Remarkably, the frequency of CD161^hi^CD8^+^ T cells was found to be the highest in patients with a low percentage of CD3^+^ T cells (data not shown) or CD8^+^ T cells, as observed for both absolute levels (data not shown) as well as for percentages (Figure 1D). In contrast, this was not observed for CD161^+^CD4^+^ T cells. Altogether, these data show that both CD161^+^CD4^+^ T cells and CD161^hi^CD8^+^ T cells reconstitute shortly after HLA-matched allo-SCT. However, while CD161^+^CD4^+^ T cells were found at higher counts compared to those of healthy controls and reconstituted to normal levels, the percentage of CD161^hi^CD8^+^ T cells declined in time due to the expansion of the CD161^neg/low^CD8^+^ T cells.

### CD161^hi^CD8^+^ T Cells are Relative Resistant to CsA Treatment

**Figure 2:**
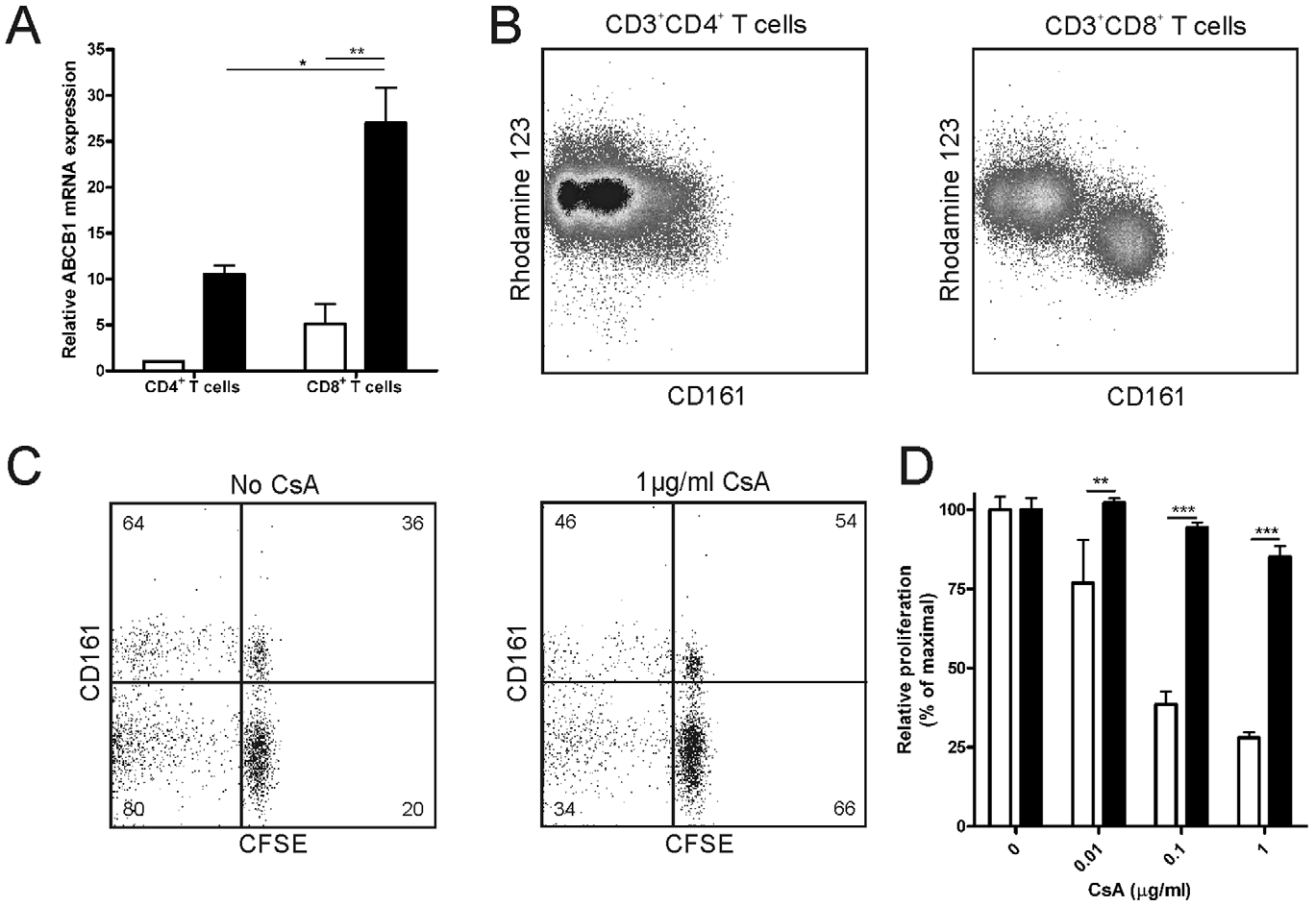
CD161^hi^CD8^+^ T cells escape from functional inhibition by CsA. (A) Relative ABCB1 expression determined by qPCR on sort-purified CD161^−^CD4^+^, CD161^neg/low^CD8^+^ (white bars) and CD161^+^CD4^+^, CD161^hi^CD8^+^ (black bars) T cells. Expression is normalized to that of CD161^−^CD4^+^ T cells. Data represent the mean ± SEM of 2 independent healthy donors. (B) PBMCs were labelled with Rh123, cultured for 30 minutes, and measured by flow cytometry for CD3, CD4, CD8, and CD161. A representative sample is shown gated on CD3^+^CD4^+^ and CD3^+^CD8^+^ T cells. (C-D) CFSE-labelled PBMCs were stimulated in an MLR with irradiated allo-PBMCs in the presence or absence of CsA. CFSE levels were determined after 7 days of culture. (C) Flow cytometry data of one representative experiment is shown gated on CD3^+^CD8^+^ T cells. Numbers represent percentages of proliferation per subset. (D) Relative proliferation is depicted as the percentage proliferating CD161^neg/low^T cells (white bars) and CD161^hi^CD8^+^ T cells (black bars) compared to the condition without CsA, as mean+SD (*n* = 3). One representative experiment out of 3 independent healthy donors is shown. Statistical analysis was performed using One way ANOVA followed by a Bonferroni post-hoc test. *P<0.05, **P<0.01, ***P<0.001.

Since the highest frequency and a specific decrease in the percentage of CD161^hi^CD8^+^ T cells was observed shortly after allo-SCT, we speculated that these cells were not affected by the immunosuppressive drugs which is routinely supplied to our patients in the first months after allo-SCT in order to prevent severe GVHD. CsA can be transported out of the cell by the ABCB1 transporter, which has been shown to be present on CD161^hi^ but not on CD161^neg/low^CD8^+^ T cells [32], [43]. To determine whether the ABCB1 transporter is also present on CD161-expressing CD4^+^ T cells, qPCR analysis was performed on mRNA of sorted CD161^−^CD4^+^, CD161^+^CD4^+^, CD161^neg/low^CD8^+^, and CD161^hi^CD8^+^ T cells. Next to the high ABCB1 mRNA expression in CD161^hi^CD8^+^ T cells (*P*<0.01), this transporter was also expressed by CD161^+^CD4^+^ T cells, though levels were lower (Figure 2A). This difference in ABCB1 mRNA expression levels correlated with the efflux of the fluorescent dye Rh123 by the various subsets (Figure 2B). Especially CD161^hi^CD8^+^ T cells displayed a more efficient Rh123-efflux compared to their CD161^neg/low^ counterparts. In contrast, CD161^+^CD4^+^ T cells, expressing lower mRNA levels of ABCB1 were hardly able to efflux Rh123.

To determine whether expression of the ABCB1 multi-drug transporter indeed mediates resistance of CD161-expressing T cells to CsA-induced functional impairment, CFSE-labelled PBMCs were stimulated in an MLR using irradiated allo-PBMCs in the presence or absence of physiological levels of CsA [44]. Indeed, CsA hardly affected CD161^hi^CD8^+^ T cell proliferation, whereas it exerted a strong dose-dependent inhibition on proliferation of CD161^neg/low^CD8^+^ T cells (*P*<0.001; Figure 2C–D). For CD4^+^ T cells, no difference in CsA-mediated inhibition was observed between CD161^+^ or CD161^−^CD4^+^ T cells (data not shown). Collectively, these data demonstrate that the presence of the ABCB1 transporter on CD161^hi^CD8^+^ T cells, but not on CD161^+^CD4^+^ T cells, confers relative resistance from CsA treatment.

### Decreased Levels of Circulating CD161-expressing T Cells are Associated with a Higher GVHD Incidence

**Figure 3:**
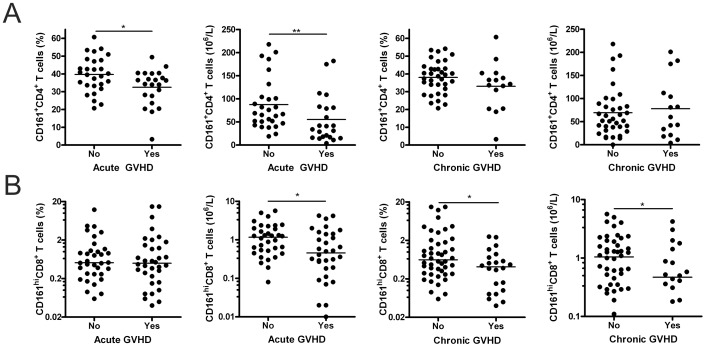
Circulating CD161^+^CD4^+^ and CD161^hi^CD8^+^ T cells are decreased in allo-SCT patients who develop GVHD. (A) Comparison of percentage and absolute levels of circulating CD161^+^CD4^+^ T cells in patients 3 months after allo-SCT and the occurrence of acute GVHD (Percentage, No *n* = 28, Yes *n* = 22; Absolute, No *n* = 28, Yes *n* = 22) and chronic GVHD (Percentage, No *n* = 35, Yes *n* = 15; Absolute, No *n* = 35, Yes *n* = 15). (B) Comparison of percentage and absolute numbers of circulating CD161^hi^CD8^+^ T cells in patients 3 months after allo-SCT and the occurrence of acute GVHD (Percentage, No *n* = 34, Yes *n* = 34; Absolute, No *n* = 34, Yes *n* = 32) and chronic GVHD (Percentage, No *n* = 46, Yes *n* = 22; Absolute, No *n* = 46, Yes *n* = 20). Lines represent mean (CD4) or median (CD8) values. Statistical analysis was performed using univariate logistic regression analysis. *P<0.05, **P<0.01.

To determine whether CD161-expressing T cells are associated with the development of GVHD, statistical analysis was performed between relative and absolute cell counts of CD161^+^CD4^+^ and CD161^hi^CD8^+^ T cells measured in the circulation of patients at 3 months after allo-SCT and their GVHD status. Both the percentage (39.7%, range 20.7–60.7 vs. 32.5%, range 3.25–49.44, *P* = 0.025 ) and absolute levels (87.5×10^6^/L, range 19–218 vs. 55.3×10^6^/L, range 4–182, *P* = 0.009) of CD161^+^CD4^+^ T cells in peripheral blood were found to be significantly reduced in patients with acute GVHD (Figure 3A). This decrease was also observed for the absolute numbers of CD161^hi^CD8^+^ T cells (1.17×10^6^/L, range 0.08–5.64, vs. 0.46×10^6^/L, range 0.01–4.21, *P = *0.013) (Figure 3B). In addition, the percentage (0.62%, range 0.06–14.80, vs. 0.41%, range 0.04–2.50, *P* = 0.019) as well as the absolute cell count (1.05×10^6^/L, range 0.07–5.64, vs. 0.47×10^6^/L, range 0.01–4.21, *P* = 0.033) of circulating CD161^hi^CD8^+^ T cells were also inversely correlated with the development of chronic GVHD (Figure 3B). Importantly, correction for known confounders like patients’ age, gender combination, and source of the graft had no or only minimal impact on the significant association between decreased CD161-expressing T cell counts and the occurrence of GVHD (Table 2). Additionally, also correction for the relative and absolute number of total CD4^+^ or CD8^+^ T cells did not affect on the observed correlation. These data indicate that reduced levels of circulating CD161^+^CD4^+^ and CD161^hi^CD8^+^ T cells correlate with an increased occurrence of GVHD.

**Table 2:** Multi-variable analysis of GVHD.

	CD161^+^CD4^+^ T cells								CD161^hi^CD8^+^ T cells									
	%		OR (95% CI)			10^6^/L		OR (95% CI)			%		OR (95% CI)		10^6^/L		OR (95% CI)	
**Acute GVHD**																		
Without correction		*P* = 0.025		0.928 (0.870–0.991)	*P* = 0.009		0.330 (0.143–0.761)			*P* = 0.811		0.958 (0.675–1.359)		*P = *0.013		0.570 (0.366–0.888)		
Corrected for																		
− Age of the patient		*P* = 0.024		0.926 (0.866–0.990)	*P* = 0.009		0.328 (0.142–0.757)			*P* = 0.678		0.927 (0.647–1.327)		*P* = 0.014		0.567 (0.362–0.890)		
− Gender combination		*P* = 0.088		0.943 (0.881–1.009)	*P* = 0.014		0.344 (0.146–0.808)			*P* = 0.798		0.950 (0.641–1.409)		*P = *0.048		0.631 (0.400–0.997)		
− Source graft		*P* = 0.027		0.929 (0.870–0.991)	*P* = 0.009		0.325 (0.140–0.754)			*P* = 0.819		0.960 (0.676–1.363)		*P* = 0.011		0.560 (0.359–0.873)		
− T cell subset (%)		*P* = 0.022		0.925 (0.865–0.989)	*P* = 0.010		0.336 (0.147–0.771)			*P* = 0.371		0.827 (0.546–1.254)		*P* = 0.021		0.567 (0.350–0.918)		
− T cell subset (10^6^/L)		*P* = 0.009		0.909 (0.846–0.976)	*P* = 0.011		0.098 (0.017–0.583)			*P* = 0.221		0.763 (0.495–1.177)		*P* = 0.025		0.577 (0.357–0.933)		
**Chronic GVHD**																		
Without correction		*P* = 0.140		0.956 (0.900–1.015)	*P* = 0.700		0.868 (0.427–1.765)			*P* = 0.019		0.588 (0.377–0.918)		*P* = 0.033		0.634 (0.418–0.963)		
Corrected for																		
− Age of the patient		P = 0.147		0.956 (0.899–1.016)	P = 0.678		0.859 (0.420–1.759)			*P* = 0.012		0.542 (0.336–0.876)		*P* = 0.035		0.635 (0.416–0.969)		
− Gender combination		P = 0.340		0.970 (0.910–1.033)	P = 0.838		0.929 (0.446–1.926)			*P = *0.012		0.473 (0.264–0.847)		*P* = 0.030		0.594 (0.372–0.951)		
− Source graft		P = 0.242		0.961 (0.899–1.027)	P = 0.612		0.816 (0.373–1.788)			*P = *0.017		0.578 (0.369–0.906)		*P* = 0.064		0.666 (0.432–1.025)		
− T cell subset (%)		P = 0.128		0.953 (0.896–1.014)	P = 0.706		0.872 (0.427–1.779)			*P = *0.006		0.473 (0.278–0.807)		*P* = 0.028		0.588 (0.366–0.943)		
− T cell subset (10^6^/L)		P = 0.251		0.964 (0.905–1.026)	P = 0.082		0.291 (0.073–1.169)			*P = *0.006		0.428 (0.235–0.781)		*P* = 0.014		0.544 (0.355–0.884)		

Abbreviations: OR, odds ratio; CI, confidence interval.

### Phenotype of CD161-expressing T Cells in Allo-SCT Patients

**Figure 4:**
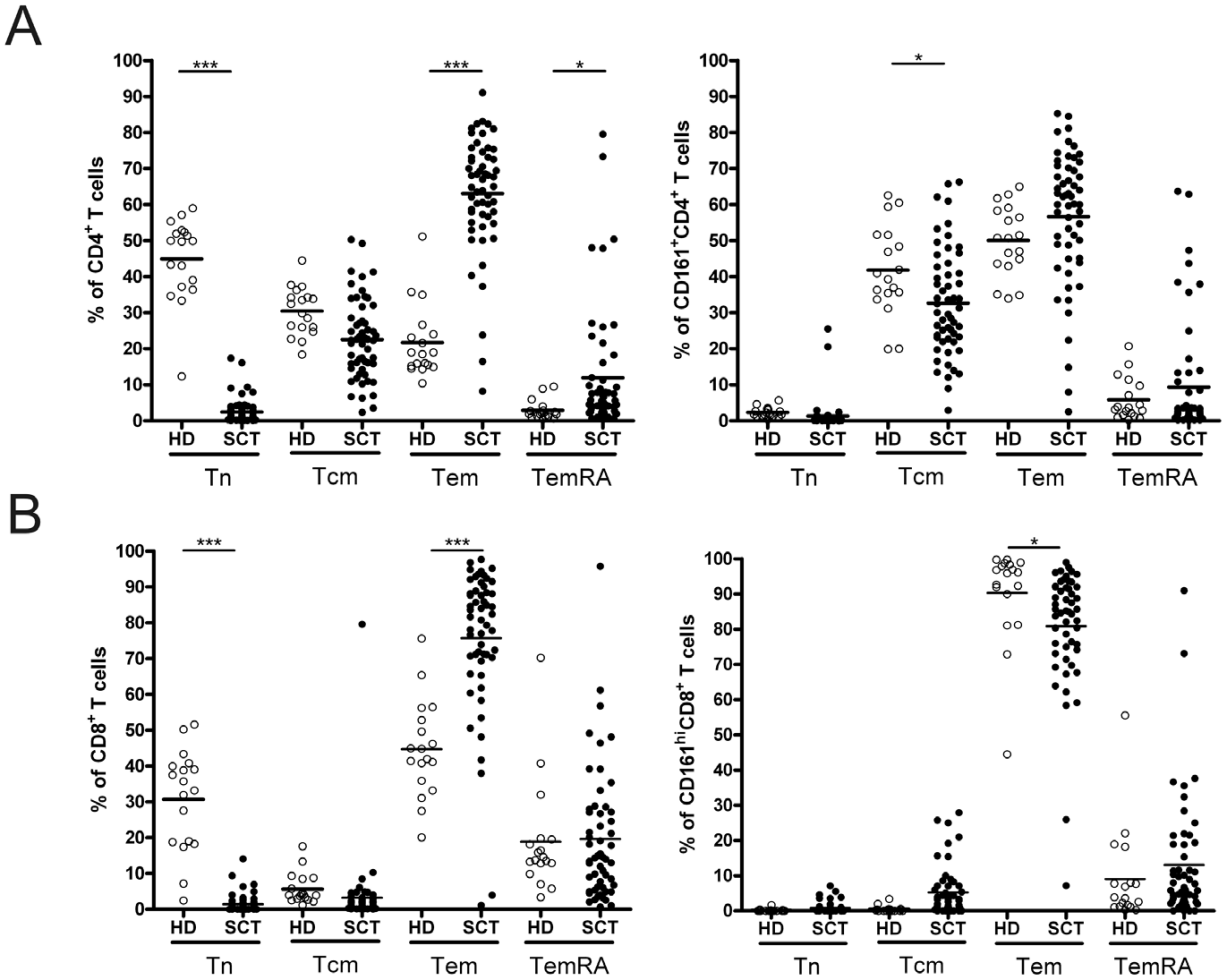
Memory phenotype of CD161-expressing T cells. (A) Memory phenotype of total CD4^+^ T cells (left panel) and CD161^+^CD4^+^ T cells (right panel) in healthy controls (○, *n* = 18) and patients 3 months after allo-SCT (•, *n* = 55). (B) Memory phenotype of total CD8^+^ T cells (left panel) and CD161^hi^CD8^+^ T cells (right panel) in healthy controls (○, *n* = 18) and patients 3 months after allo-SCT (•, *n* = 55 and *n* = 52 respectively). Tn, naïve (CCR7^+^CD45RA^+^); Tcm, central memory (CCR7^+^CD45RA^-^); Tem, effector memory (CCR7^−^CD45RA^-^); TemRA, CD45RA^+^ effector memory (CCR7^−^CD45RA^+^). Statistical analysis was performed using One-way ANOVA followed by a Bonferroni post-hoc test. *P<0.05, ***P<0.001.

To define the memory phenotype of CD161-expressing T cells in allo-SCT patients versus healthy controls, T cell subsets were analysed for the expression of CCR7 and CD45RA. In allo-SCT patients, CD4^+^ and CD8^+^ T cells have an increased proportion of effector memory cells (Tem) compared to healthy donors (Figure 4A–B, *P*<0.001). Only minimal differences were found in the differentiation status within the CD161-expressing T cell subsets between allo-SCT patient and healthy donors. While CD161^+^CD4^+^ T cells displayed both a central memory (Tcm) as well as a Tem phenotype, CD161^hi^CD8^+^ T cells consisted mainly of Tem cells. Overall, CD161-expressing T cells in allo-SCT patients, in both the CD4^+^ and CD8^+^ T cell population, seemed to be a bit more differentiated in their memory phenotype compared to healthy controls.

### CD161-expressing T Cells in Allo-SCT Patients Contain High Frequency of IL17- and IFNγ-producers

**Figure 5:**
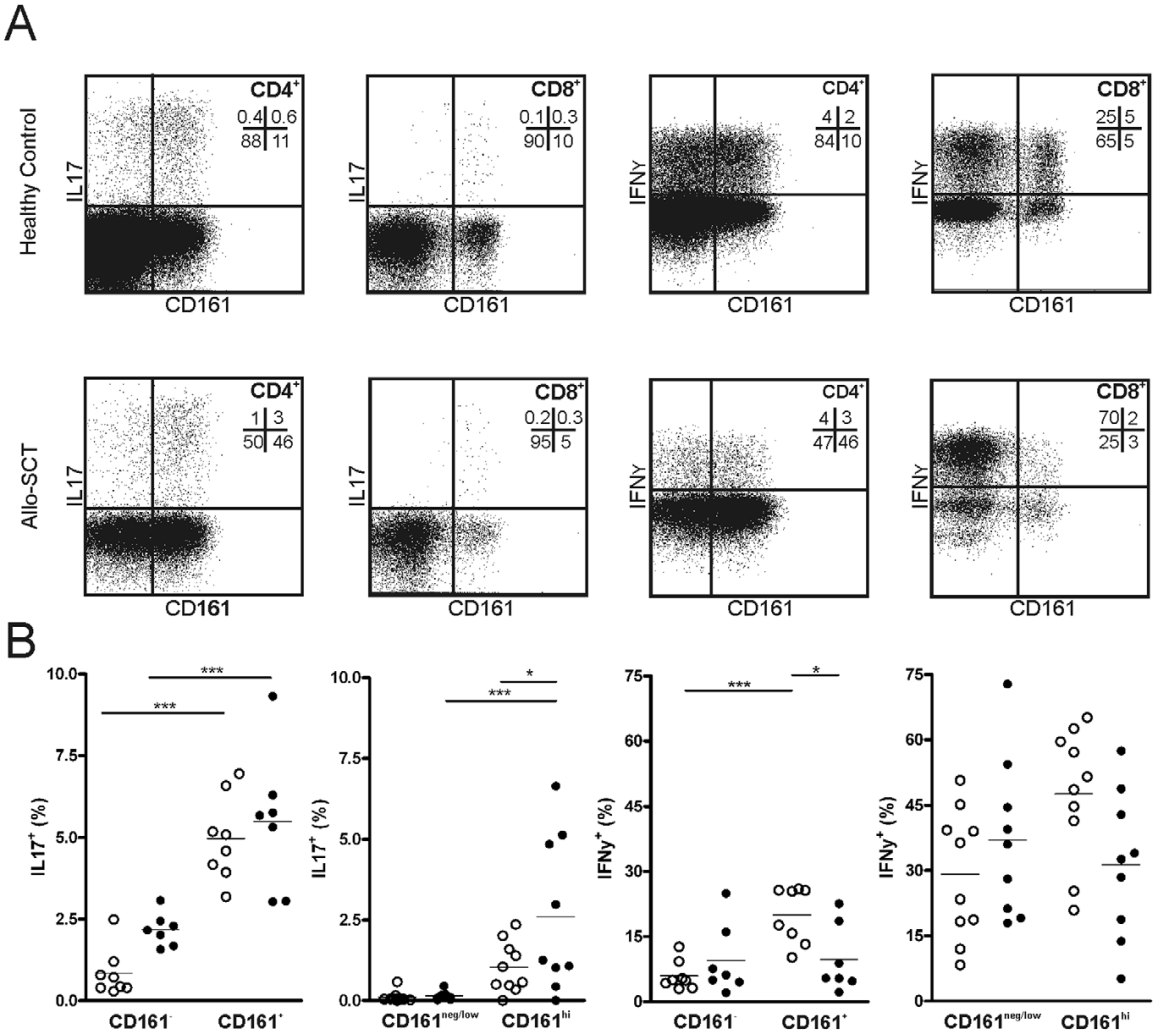
IL17 and IFNγ production of CD161-expressing T cells after allo-SCT. (A) Representative IL17 and IFNγ production by intracellular cytokine staining from a healthy control and allo-SCT patient. Numbers plot indicate the percentage of cells in each quadrant. (B) Comparison of IL17 (left panels) and IFNγ (right panels) production, measured by intracellular cytokine staining, between CD161^−^ and CD161^+^CD4^+^ T cells, and CD161^neg/low^ and CD161^hi^CD8^+^ T cells in healthy controls (○) and patients 1–3 months after allo-SCT (•). Data is shown as the percentage of positive cells, lines represent mean value. Statistical analysis was performed using One-way ANOVA followed by a Bonferroni post-hoc test, or a one-tailed paired student t-test. *P<0.05, ***P<0.001.

Next, functionality of CD161-expressing T cells was investigated by defining their cytokine production profile. Therefore, PBMCs from both healthy controls as well as allo-SCT patients were stimulated with PMA and ionomycin and subsequently stained for intracellular cytokines (Figure S2). Both CD161^+^CD4^+^ as well as CD161^hi^CD8^+^ T cells showed to be dominant producers of IL17 within their T cell compartment (Figure 5A–B, *P*<0.001). Interestingly, for CD8^+^ T cells the percentage of IL17-secreting cells within the CD161-expressing cells was higher in allo-SCT patients compared to healthy controls (*P*<0.05). In addition, as compared to CD161^−^CD4^+^ T cells, the frequency of IFNγ-producers was increased in CD161^+^CD4^+^ T cells of healthy controls, but not for allo-SCT patients (Figure 5A–B, *P*<0.001). Together, these data demonstrate that CD161-expressing T cells of allo-SCT patients contain a high frequency of IFNγ- and IL17-producers.

### CCR6-mediated Migration of CD161-expressing T Cells to CCL20 Present in GVHD Tissues

**Figure 6:**
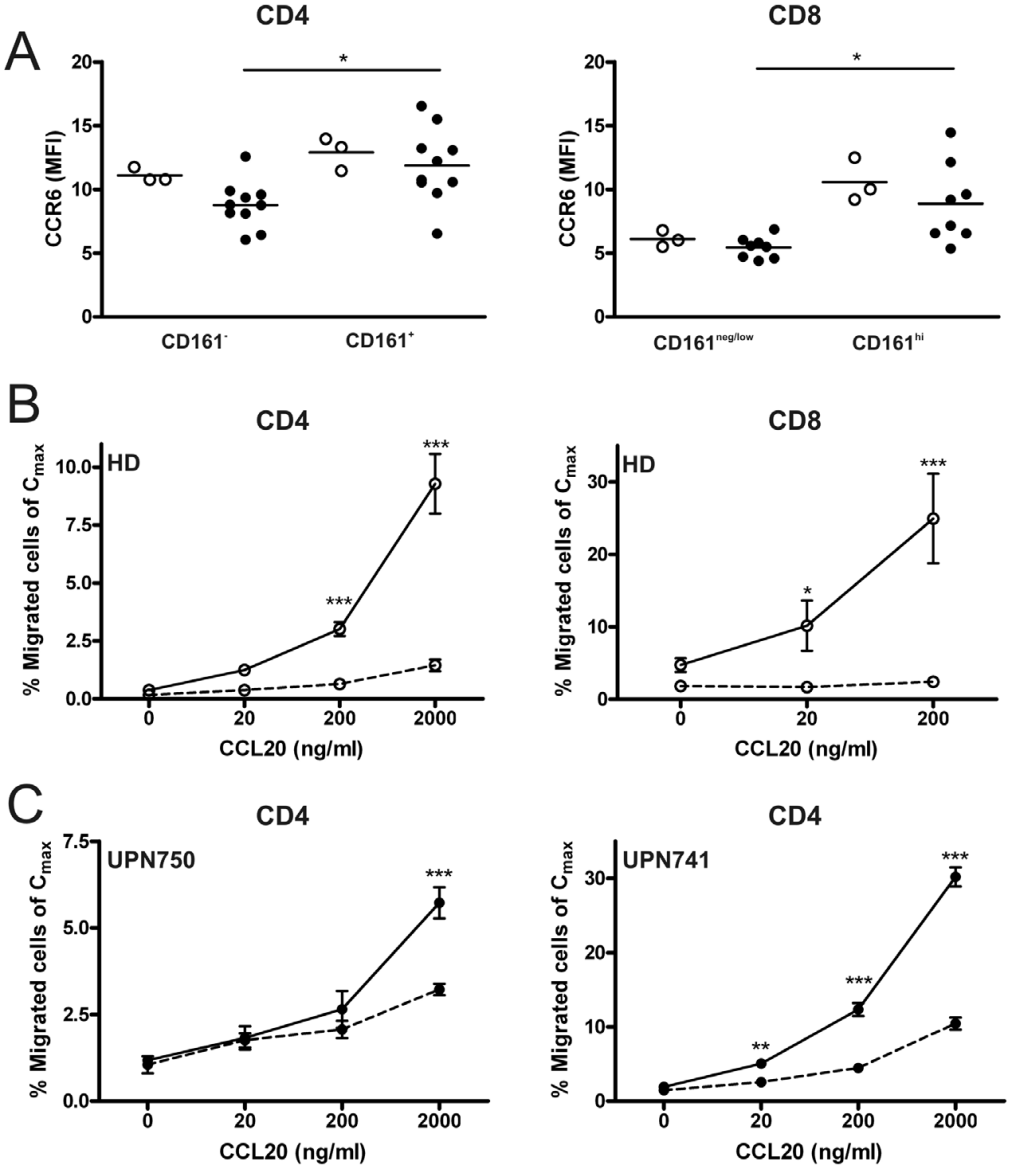
Preferential migration of CD161-expressing T cells to CCL20. (A) Comparison of CCR6 expression between CD161^−^ and CD161^+^CD4^+^ memory T cells, and CD161^neg/low^ and CD161^hi^CD8^+^ Tem cells of healthy donors (о) and patients 3 months after allo-SCT (•). The frequency of CD161^+^CD4^+^ T cells in the measured samples was 34.6–55.7% in allo-SCT patients (*n* = 10) and 36.6–46.4% in healthy controls (*n* = 3), and the frequency of CD161^hi^CD8^+^ T cells was 0.05–23.8% in allo-SCT patients (*n* = 8) and 11.5–42.4% in healthy controls (*n* = 3). Data is shown as mean fluorescent intensity (MFI). Lines represent mean value. (B) Migration of CD4^+^ and CD8^+^ T cell subsets to CCL20. CD4^+^ or CD8^+^ T cells were applied to the upper chamber and migrated to the lower chamber containing medium with or without CCL20. Migrated cells were analysed by flow cytometry. Migration is shown relative to input (mean ± SD) for CD161^−^CD4^+^ and CD161^neg/low^CD8^+^ T cells (dashed line), and CD161^+^CD4^+^ and CD161^hi^CD8^+^ T cells (solid line) (*n* = 3). One representative experiment out of 2 (CD4) or 3 (CD8) independent healthy donors is shown. (C) Migration of CD4^+^ T cell subsets to CCL20. CD3^+^ T cells were applied to the upper chamber and migrated to the lower chamber containing medium with or without CCL20. Migrated cells were analysed by flow cytometry. Migration is shown relative to input (mean ± SD) for CD161^−^CD4^+^ (dashed line), and CD161^+^CD4^+^ (solid line) (n = 3). Two independent patients 3 months after allo-ST are shown. Statistical analysis was performed using Two-way ANOVA followed by a Bonferroni post-hoc test. *P<0.05, **P<0.01, ***P<0.001.

To explore the tissue-homing capacity of CD161-expressing T cells in allo-SCT patients, we determined expression of the chemokine receptor CCR6, within the corresponding memory-phenotype. CCR6 expression was found to be elevated on both CD161^+^CD4^+^ as well as CD161^hi^CD8^+^ T cells compared to their CD161-negative counterparts (Figure 6A and Figure S3, *P*<0.05). Next, the migration potential of CCR6^+^CD161-expressing T cells towards CCL20, the sole ligand for CCR6, was determined in an *in vitro* migration assay using either purified CD4^+^ or CD8^+^ T cells isolated from healthy controls. Where CCL20 induced robust migration of both CD161^+^CD4^+^ as well as CD161^hi^CD8^+^ T cells, their CD161-negative counterparts were unresponsive to this chemokine (Figure 6B; *P*<0.001). In addition, migration assays were performed using purified CD3^+^ T cells from allo-SCT patients. Since CD8^+^ T cells numbers were too low, analysis was focused on CD4^+^ T cells. For these, specific migration of CD161^+^CD4^+^ T cells towards CCL20 was observed compared to CD161^−^CD4^+^ T cells (Figure 6C; *P*<0.001). These results support the concept that CCR6 expression on CD161^+^CD4^+^ and CD161^hi^CD8^+^ T cells facilitates preferential migration to CCL20-expressing tissues.

**Figure 7:**
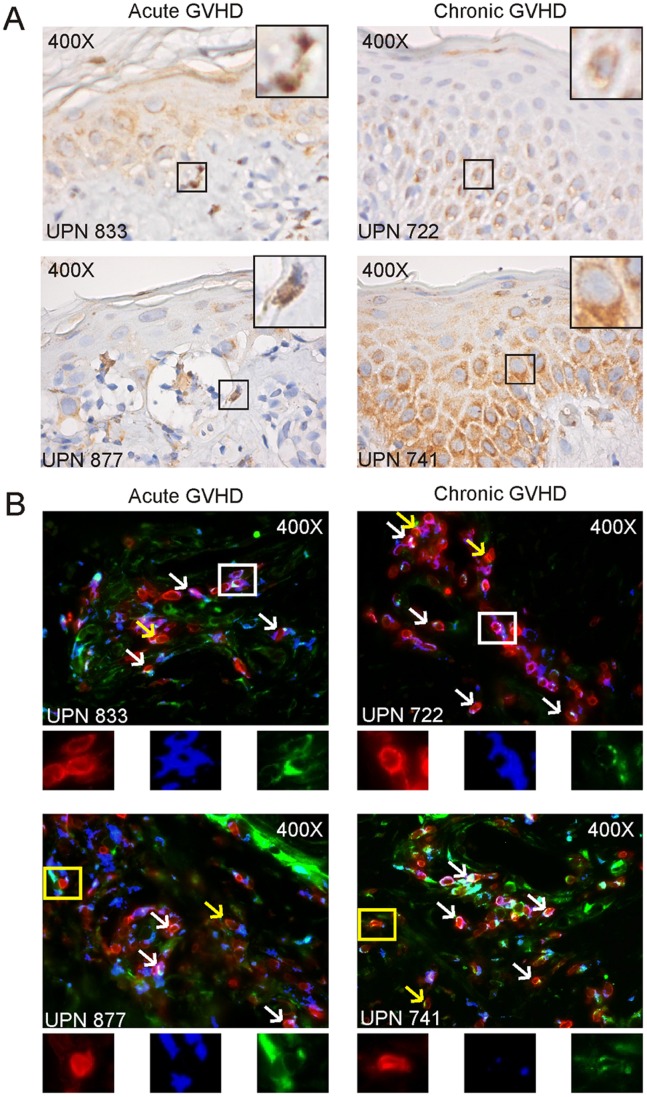
CCL20 is expressed in GVHD tissues and selectively attracts CCR6^+^ T cells. (A) CCL20 staining in skin biopsies of patients diagnosed with acute GVHD at respectively 49 (UPN 833) and 30 days (UPN 877), or chronic GVHD 127 days (UPN 722) and 321 days (UPN 741) days after allo-SCT. Squares indicate examples of single cells in the dermis in close proximity to the epidermal-dermal junction (acute GVHD) or in the epidermis which are situated in close proximity to or at the epidermal-dermal junction (chronic GVHD). Images were captured at 400X. (B) CD3 (red), CD4 (blue), and CCR6 (green) triple staining in skin biopsies of patients diagnosed with acute GVHD at respectively 49 (UPN 833) and 30 days (UPN 877), or chronic GVHD 127 days (UPN 722) and 321 days (UPN 741) days after allo-SCT. White arrows and squares indicate examples of CD3^+^CD4^+^CCR6^+^ cells, yellow arrows and squares indicate examples of CD3^+^CD4^−^CCR6^+^ cells. Single stainings of the cells in squares are depicted under the image. Images were captured at 400X.

To further substantiate the relevance of the CCR6-CCL20 pathway in guiding migration of CD161-expressing T cells to GVHD-prone organs, we next determined CCL20 expression *in situ* in skin biopsies obtained from allo-SCT recipients who developed acute or chronic GVHD (Figure 7A, Figure S4). CCL20 expression could be clearly visualized in the epidermis of the skin. Moreover, CCL20 was also expressed on single cells present in the dermis in close proximity to the epidermal-dermal junction that is typically targeted by infiltrating T cells.

Finally, to confirm homing of CD161^+^CCR6^+^ co-expressing T cells to GVHD tissues, skin biopsies of GVHD patients were examined for the presence of CCR6^+^ T cells. Infiltrated T cells were clearly visible in the skin derived from both acute as well as chronic GVHD patients, of which several cells displayed expression of CCR6 (Figure 7B). Among these cells, both CD3^+^CD4^+^ as well as CD3^+^CD4^−^ T cells were detected. Also in GVHD-affected gut tissue, expression of CCL20 and CCR6^+^CD3^+^ T cells could be detected (Figure S5). These data strengthen our hypothesis that the observed decrease of CD161-expressing T cells from the circulation in GVHD patients is the result of their homing to CCL20-expressing GVHD tissues.

## Discussion

Th17 cells have been linked to the pathophysiology of GVHD, however data remains limited and contradictory [8]–[15]. Recently, correlations have been made between Th17 and Tc17 cells and their involvement in GVHD using the well-established CD161 lineage-identifier for IL17-producing CD4^+^ and CD8^+^ T cells subsets [16], [18], [19], [26]. The involvement of CD161-expressing T cells is further supported by their responsiveness to IL1ß [26], a key cytokine involved in GVHD [1], [45]. In this study, we investigated the repopulation kinetics of both CD4^+^ and CD8^+^ CD161-expressing T cells in allo-SCT patients, and their potential role in the development of GVHD. Remarkably, the relative proportion of CD161^+^ cells within the CD4^+^ T cells compartment was higher in allo-SCT patients compared to healthy controls. Furthermore, while CD161^+^CD4^+^ T cells reconstituted allo-SCT patients in a relatively balanced way, we found that the CD161^hi^CD8^+^ T cell frequency decreases within the first 3 months after allo-SCT, where after it remained stable at a low level. Interestingly, this relative decrease occurred during tapering of CsA, which is normally started two months after allo-SCT when GVHD is absent. During these first months after allo-SCT, CD161^hi^CD8^+^ T cells, expressing the ABCB1 transporter, have a proliferative advantage over CD161^neg/low^CD8^+^ T cells as evidenced by their clear *in vitro* resistance to the CsA-mediated inhibition of T cell proliferation. During tapering of CsA, this advantage gradually disappeared, which could explain the rise of CD161^neg/low^CD8^+^ T cells and a relative decline of CD161^hi^CD8^+^ T cells. Furthermore, it has been described that CD161^hi^CD8^+^ T cells have a lower proliferation capacity compared to CD161^neg/low^CD8^+^ T cells [31], which might result in a more robust expansion of CD161^neg/low^CD8^+^ T cells at later time points after allo-SCT. Additionally, cell death as well as the administration of various drugs, like steroids for the treatment of GVHD, will also influence the repopulation kinetics shortly after allo-SCT. However, it is likely that the ABCB1-dependent CsA-efflux favours early repopulation of CD161^hi^CD8^+^ T cells following allo-SCT.

In this current study, we are the first to show a correlation between reduced levels of circulating CD161^+^CD4^+^ and CD161^hi^CD8^+^ T cells and the occurrence of GVHD. Interestingly, a significant decreased frequency was observed in PB samples 3 months after allo-SCT from patients who had acute GVHD or who developed chronic GVHD later on. This information is of considerable significance as GVHD, of which the pathogenesis is still not fully understood, is the most profound adverse complication after allo-SCT [1], [46].

Although the number of patients analysed in this study did not allow statistical analysis for multiple confounding parameters at once, we found that the association between decreased CD161-expressing T cells and GVHD remained independent from the total CD4^+^ or CD8^+^ T cell number and known confounders such as age, gender combination and graft source. Due to insufficient data, we were unable to take CsA blood levels along as a confounder in this study. However, if CsA levels in the patients’ blood would have interfered with the correlation of CD161-expressing T cells and GVHD, it is expected that higher CsA levels in GVHD patients would result in higher levels of CD161-expressing T cells due to their CsA resistance. Therefore, the found correlation between decreased levels of CD161-expressing T cells and GVHD would even be an underestimation.

Based on the found association, we speculated that the decrease in circulating CD161-expressing T cells results due to specific recruitment into GVHD-affected tissues. Prevalent expression of CCR6, as well as the migration potential of both CD161^+^CD4^+^ cells and CD161^hi^CD8^+^ T cells to inflamed tissues, and their involvement in several inflammatory disorders have been described previously [25], [28], [30], [35]. Furthermore, our observations are in agreement with the recent study of Bossard *et al.* [16], who observed increased numbers of CD161^+^, RORγt^+^ and CCR6^+^ cells in GVHD-affected intestinal mucosa. Therefore, our findings support the pathogenic role of CD161^+^CCR6^+^ co-expressing T cells, preferentially containing Th17 and Tc17 cells, in GVHD developing after allo-SCT.

Whether CD161-expressing T cells are directly involved in the onset and/or aggravation of GVHD remains to be proven. However, IL1α as well as IL1ß, which are part of the cytokine storm created after conditioning of the patient [5], [45], could result in elevated CCL20 production in GVHD-prone tissues. This possibility was shown by culturing endometriotic stromal cells in the presence of IL1ß, which increased their CCL20 secretion considerably [35]. Also damage to the skin, as well as culture of keratinocytes with IL1α increases CCL20 secretion [36]. Additionally, in agreement with other studies we have found that CD161^+^CD4^+^ and CD161^hi^CD8^+^ T secrete IFNγ and IL17 [18], [22], [26], [27], [30]. Interestingly, the IL17 production of CD161-expressing T cells is further increased after culturing these cells with IL1ß alone or in conjunction with IL23 [18], [26]. Migration of CCR6^+^CD161-expressing T cells to the GVHD-prone tissues could thereby result in IL17 production, priming the GVHD response, further enhancing CCL20 production, and recruiting other effector cells. Our data indicate that the decrease in percentage of CD161^hi^CD8^+^ T cells, already at 3 months after allo-SCT, could be an early sign of the clinical onset of chronic GVHD later on. All together, these data suggest that CD161-expressing CD4^+^ and CD8^+^ T cells with IL17 and/or IFNγ secreting properties, are involved in the onset of GVHD.

In conclusion, here we show that following allo-SCT, patients are readily reconstituted with CD161-expressing T cells. Interestingly, the resistance of CD161^hi^CD8^+^ T cells for CsA-mediated inhibition of proliferation might explain their observed repopulation kinetics. Most importantly, we observed that a decrease of both circulating CD161^+^CD4^+^ as well as CD161^hi^CD8^+^ T cells coincided with the occurrence of both acute and chronic GVHD. In fact, we demonstrate that the reduced number of CD161-expressing T cells could be seen as an independent predictor of GVHD, and that CCR6^+^CD161-expressing T cells may be involved in the immune pathology of GVHD following their CCL20-dependent recruitment into affected tissues.

## Supporting Information

Figure S1 **CD161^+^CD4^+^ and CD161^hi^CD8^+^ T cells in healthy adults.** (A) Flow cytometry data of a representative healthy control gated on circulating CD3^+^ cells. CD161^−^CD4^+^ and CD161^+^CD4^+^ T cell populations are depicted in the gates. (B) Percentage and absolute numbers of circulating CD161^+^ within CD4^+^ T cells in healthy adults (*n* = 16). (C) Flow cytometry data of a representative healthy control gated on CD3^+^ cells. CD161^neg/low^CD8^+^ and CD161^hi^CD8^+^ T cell populations are depicted in the gates. (D) Percentage and absolute levels of circulating CD161^hi^ within CD8^+^ T cells in healthy adults (*n* = 42, *n* = 22). Lines represent the mean value.
(TIF)

Figure S2 **IL17 and IFNγ production determined by intracellular staining.** Gating strategy of intracellular cytokine staining for IL17 and IFNγ 4 h after stimulation with PMA and ionomycin.
(TIF)

Figure S3 **CCR6 is higher expressed on CD161-expressing T cells.** Representative flow data of CCR6 expression on CD161-expressing CD4^+^ or CD8^+^ T cells in a patient after allo-SCT. Numbers represent the mean fluorescent intensity of the subset.
(TIF)

Figure S4 **CCL20 expression in GVHD affected skin tissue.** (A) Negative (without CCL20-specific antibody) and positive (B) control staining for CCL20 in foreskin. (C) CCL20 staining in skin biopsies of two additional patients, conditioned with Cyclo-ATG-Bus (cyclophosphamide-antithymocyte globulin-busilvex) and Flu-Mel-Alem (fludarabine-melphalan-alemtuzumab), who were diagnosed with acute GVHD at respectively 41 (UPN 1001) and 18 (UPN 10019) days after allo-SCT. Squares indicate examples of single cells in the epidermis which are situated in close proximity to the epidermal-dermal junction. Images were captured at 400X.
(TIF)

Figure S5 **CCL20 expression and CCR6^+^ T cells in GVHD affected gut tissue.** (A) CCL20 (brown) and (B) CD3 (red), CD4 (blue), and CCR6 (green) triple staining in a gut biopsy of a patient after allo-SCT diagnosed with acute GVHD at 34 day after allo-SCT (UPN 902). White arrows and squares indicate examples of CD3^+^CD4^+^CCR6^+^ cells, yellow arrows and squares indicate examples of CD3^+^CD4^−^CCR6^+^ cells. Single stainings of the cells in squares are depicted under the image. Images were captured at 400X.
(TIF)
