# Supplementary figures and images for: Decreased Levels of Circulating IL17-Producing CD161+CCR6+ T Cells Are Associated with Graft-versus-Host Disease after Allogeneic Stem Cell Transplantation
Source: PLoS One. 2012 Dec 4;7(12):e50896. doi: 10.1371/journal.pone.0050896 (PMC3514180; doi:10.1371/journal.pone.0050896)

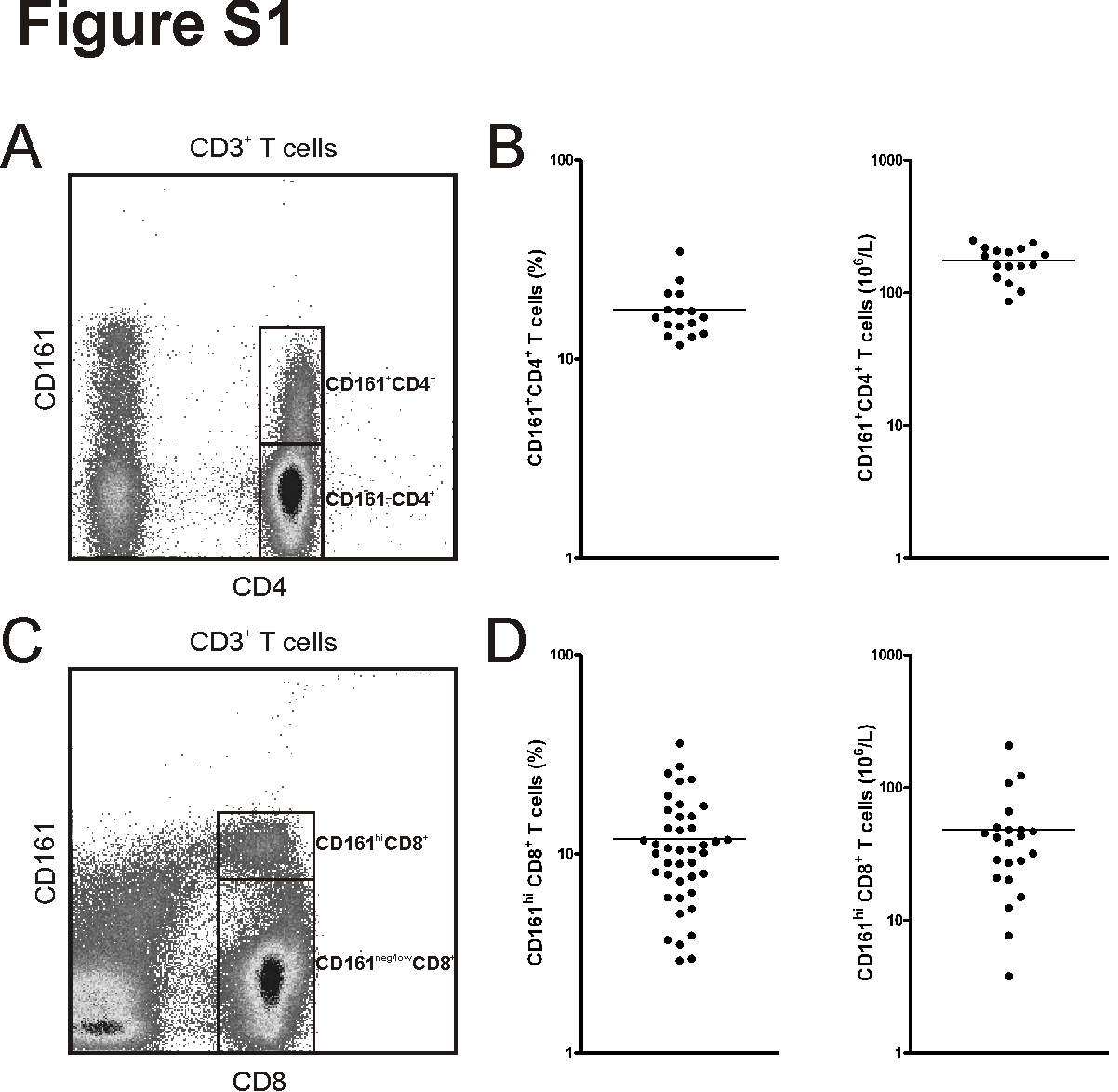

Supplement: Figure S1 — CD161+CD4+ and CD161hiCD8+ T cells in healthy adults. (A) Flow cytometry data of a representative healthy control gated on circulating CD3+ cells. CD161−CD4+ and CD161+CD4+ T cell populations are depicted in the gates. (B) Percentage and absolute numbers of circulating CD161+ within CD4+ T cells in healthy adults (n = 16). (C) Flow cytometry data of a representative healthy control gated on CD3+ cells. CD161neg/lowCD8+ and CD161hiCD8+ T cell populations are depicted in the gates. (D) Percentage and absolute levels of circulating CD161hi within CD8+ T cells in healthy adults (n = 42, n = 22). Lines represent the mean value. (TIF) [file pone.0050896.s001.tif]

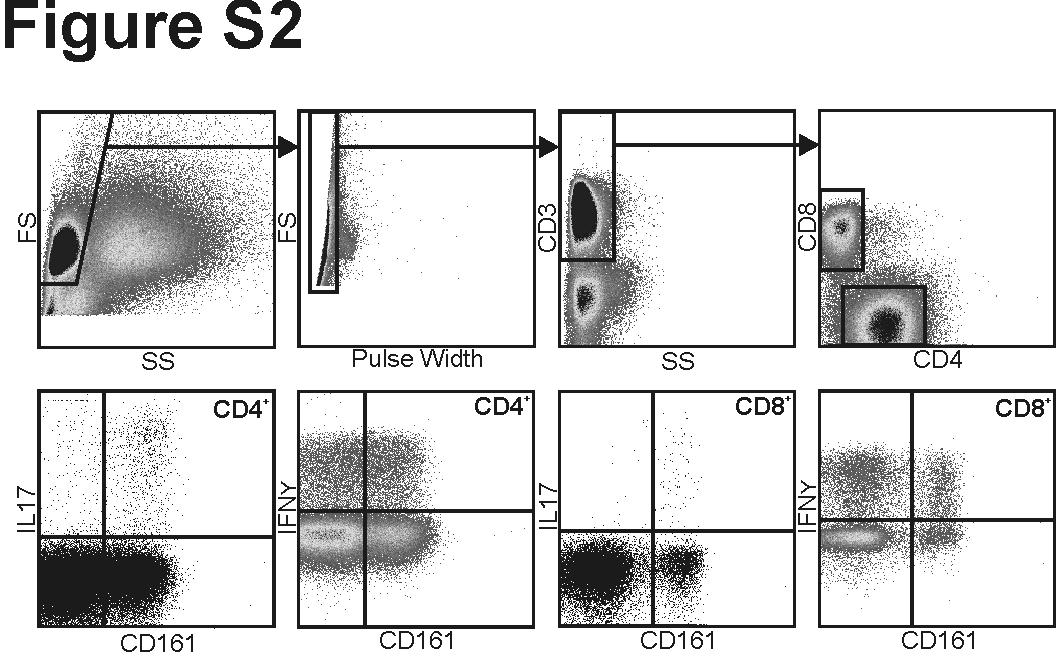

Supplement: Figure S2 — IL17 and IFNγ production determined by intracellular staining. Gating strategy of intracellular cytokine staining for IL17 and IFNγ 4 h after stimulation with PMA and ionomycin. (TIF) [file pone.0050896.s002.tif]

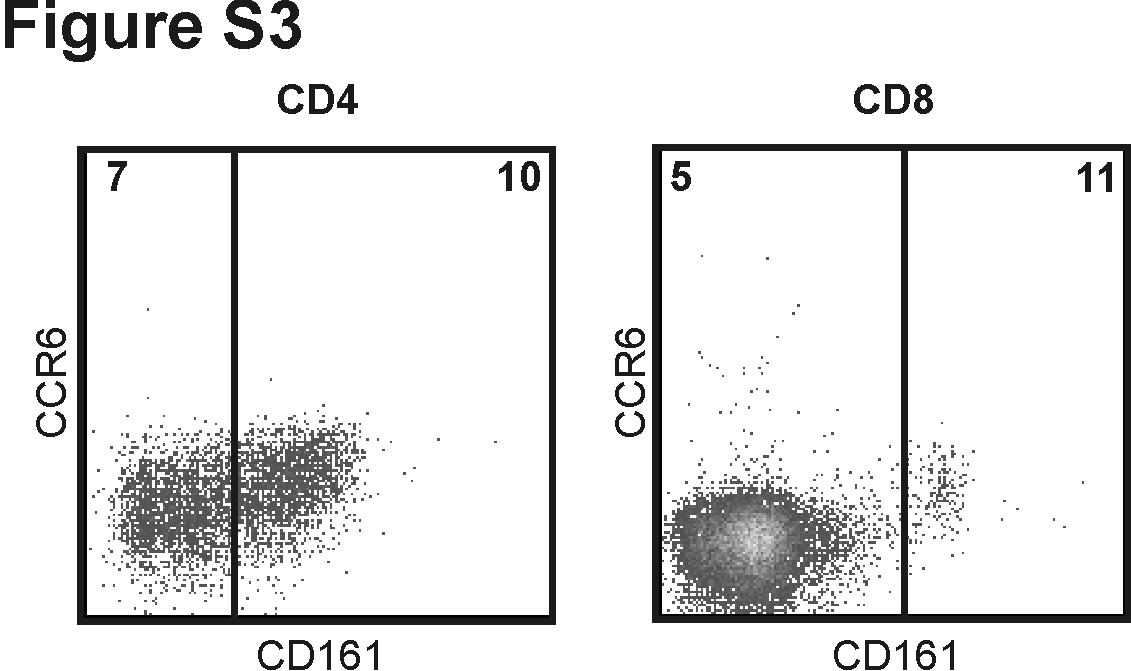

Supplement: Figure S3 — CCR6 is higher expressed on CD161-expressing T cells. Representative flow data of CCR6 expression on CD161-expressing CD4+ or CD8+ T cells in a patient after allo-SCT. Numbers represent the mean fluorescent intensity of the subset. (TIF) [file pone.0050896.s003.tif]

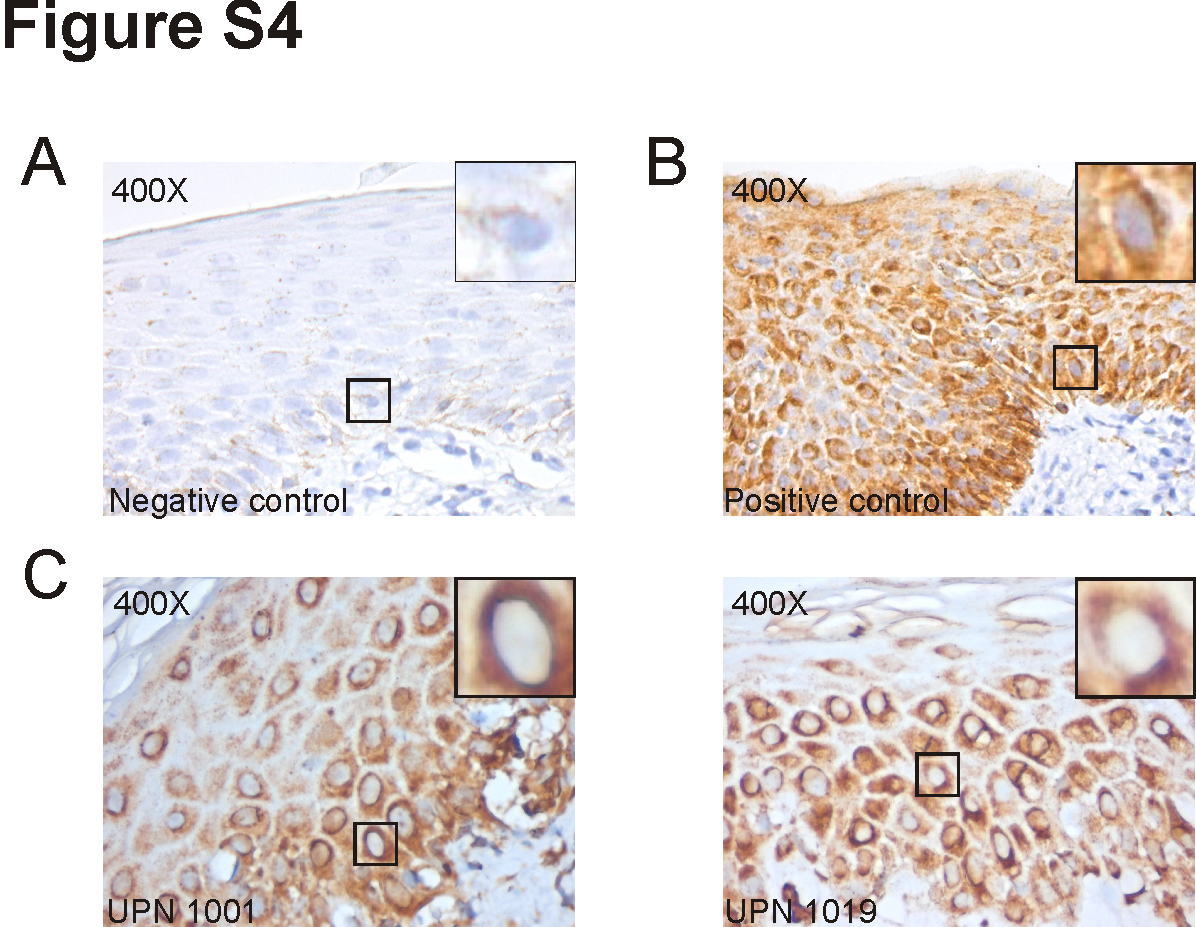

Supplement: Figure S4 — CCL20 expression in GVHD affected skin tissue. (A) Negative (without CCL20-specific antibody) and positive (B) control staining for CCL20 in foreskin. (C) CCL20 staining in skin biopsies of two additional patients, conditioned with Cyclo-ATG-Bus (cyclophosphamide-antithymocyte globulin-busilvex) and Flu-Mel-Alem (fludarabine-melphalan-alemtuzumab), who were diagnosed with acute GVHD at respectively 41 (UPN 1001) and 18 (UPN 10019) days after allo-SCT. Squares indicate examples of single cells in the epidermis which are situated in close proximity to the epidermal-dermal junction. Images were captured at 400X. (TIF) [file pone.0050896.s004.tif]

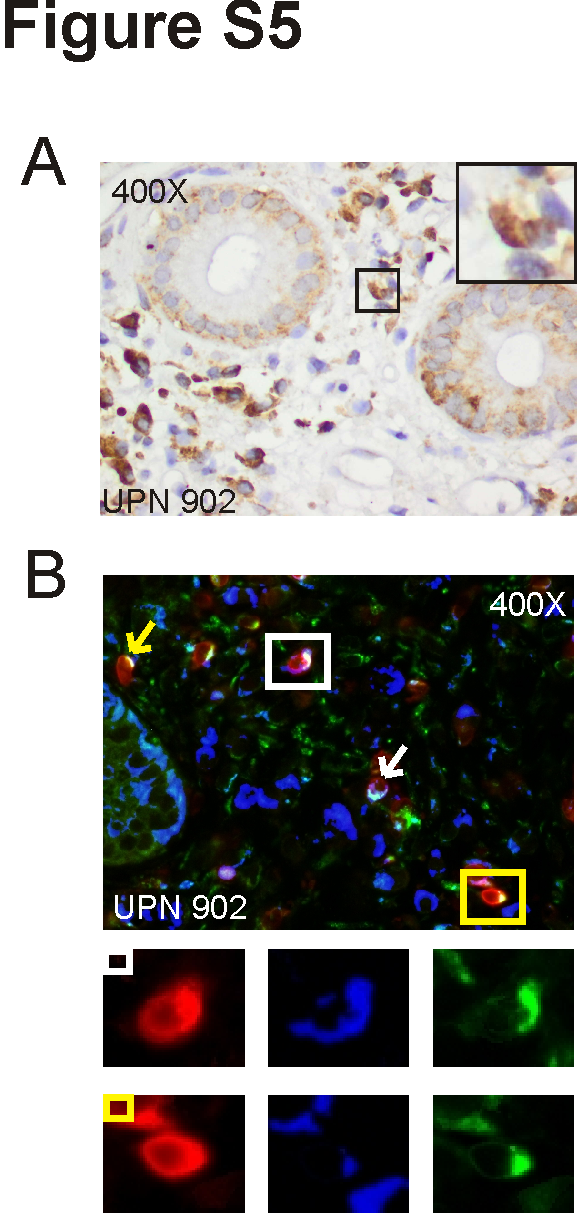

Supplement: Figure S5 — CCL20 expression and CCR6+ T cells in GVHD affected gut tissue. (A) CCL20 (brown) and (B) CD3 (red), CD4 (blue), and CCR6 (green) triple staining in a gut biopsy of a patient after allo-SCT diagnosed with acute GVHD at 34 day after allo-SCT (UPN 902). White arrows and squares indicate examples of CD3+CD4+CCR6+ cells, yellow arrows and squares indicate examples of CD3+CD4−CCR6+ cells. Single stainings of the cells in squares are depicted under the image. Images were captured at 400X. (TIF) [file pone.0050896.s005.tif]
